# Hot and Cold Drinks

**Published:** 1883-01

**Authors:** 


### HOT AND COLD DRINKS.

A correspondent of *Knowledge* calls attention to some of the disadvantages of hot drinks. Cold drinks, he says, are natural to man, though most people now-a-days are so used to hot drinks that they do not feel satisfaction—really stimulation—unless they have them. Hot drinks are injurious to the tongue, for they deaden its sensation, and, after taking hot soup or drink, the tongue becomes quite numb, and unable to taste the finer flavors of a dish. The teeth are greatly injured by them, and many dentists say caries (decay) is due to them alone. They crack the enamel, and thus allow caries to set in. When caries has once set in, hot drinks are a common cause of neuralgia.

Hot drinks are specially hurtful to the stomach. They cause irritation of the nerves of the stomach and consequent mild inflammation of that organ, so that after a hot drink the stomach is red and congested ; in time a debilitated condition is set up. A temperature of 100 degrees Fahrenheit also destroys the active ferment of the gastric juice—pepsin—and so leads to indigestion. If the stomach is at all disordered, hot drinks give rise to much griping pain, and in many cases to vomiting.

In cases of diarrhœa, too, hot drinks only increase it, while cold ones tend to lessen it. Thirst is not common in winter, unless sugary, salty or hot-spiced foods have been taken. In cold weather the air contains more moisture than in hot, and in cold weather there is less perspiration. Hot drinks increase the volume of heat in the body, and if that is not required, it is quickiy got rid of by the skin. Water is the best thirst quencher, but if simple food be taken the need of drinks will be small. Many vegetarians drink nothing from month to month, the only fluid they get being the juices of the fruits which they eat. But pleasant drinks, like tea, coffee, etc., may be taken lukewarm for a long time with little apparent damyge. The least injurious is cocoa, made with plenty of milk, and allowed to stand until nearly cool. A good test is to apply the little finger to the drink, and if it be not to it, then it may be safely taken.

